# Using Human Plasma as an Assay Medium in Caco-2 Studies Improves Mass Balance for Lipophilic Compounds

**DOI:** 10.1007/s11095-018-2493-3

**Published:** 2018-09-17

**Authors:** Kasiram Katneni, Thao Pham, Jessica Saunders, Gong Chen, Rahul Patil, Karen L. White, Nada Abla, Francis C. K. Chiu, David M. Shackleford, Susan A. Charman

**Affiliations:** 1 Centre for Drug Candidate Optimisation, Monash Institute of Pharmaceutical Sciences, 381 Royal Parade, Parkville, VIC 3052 Australia; 2 Medicines for Malaria Venture, 20, Route de Pré-Bois, 1215 Geneva 15, Switzerland

**Keywords:** Caco-2 permeability, human plasma protein binding, lipophilic compounds

## Abstract

**Purpose:**

To examine the utility of human plasma as an assay medium in Caco-2 permeability studies to overcome poor mass balance and inadequate sink conditions frequently encountered with lipophilic compounds.

**Methods:**

Caco-2 permeability was assessed for reference compounds with known transport mechanisms using either pH 7.4 buffer or human plasma as the assay medium in both the apical and basolateral chambers. When using plasma, P_app_ values were corrected for the unbound fraction in the donor chamber. The utility of the approach was assessed by measuring the permeability of selected antimalarial compounds using the two assay media.

**Results:**

Caco-2 cell monolayer integrity and P-gp transporter function were unaffected by the presence of human plasma in the donor and acceptor chambers. For many of the reference compounds having good mass balance with buffer as the medium, higher P_app_ values were observed with plasma, likely due to improved acceptor sink conditions. The lipophilic antimalarial compounds exhibited low mass balance with buffer, however the use of plasma markedly improved mass balance allowing the determination of more reliable P_app_ values.

**Conclusions:**

The results support the utility of human plasma as an alternate Caco-2 assay medium to improve mass balance and permeability measurements for lipophilic compounds.

**Electronic supplementary material:**

The online version of this article (10.1007/s11095-018-2493-3) contains supplementary material, which is available to authorized users.

## Introduction

*In vitro* permeability methods based on cell-based systems (e.g. Caco-2 and MDCK), isolated tissues, or artificial membranes (PAMPA) are well-established for testing the likely absorption properties of new drug candidates (1). Among these, the Caco-2 cell monolayer system has been widely adopted based on positive correlations between apparent permeability coefficients (P_app_) determined using this model and human jejunal permeability (2,3). Since fully differentiated Caco-2 cells express a number of transporters found in human enterocytes (4), assessment of bidirectional Caco-2 permeability can also be used to identify whether or not compounds are inhibitors and/or substrates for common transporters. As such, the Caco-2 model is recommended by the FDA and EMA as one of the cell-based screening strategies to inform the need for *in vivo* transporter-based drug-drug interaction studies (5,6). Permeability estimates are also important input parameters for in silico prediction of compound absorption via physiologically-based pharmacokinetic (PBPK) models, and the FDA acknowledges the use of Caco-2 and other cultured epithelial cell monolayer test systems to assess permeability in relation to the biopharmaceutics classification system (BCS) and associated bio-waiver applications (7,8). Compound permeability is also a key component of the biopharmaceutics drug disposition classification system (BDDCS) (9), the extended clearance classification system (ECCS) (10), and the developability classification system (DCS) (11), each of which can be used to tailor compound profiling procedures during drug discovery and development.

Determination of P_app_ values is most frequently based on the measured steady-state rate of appearance of compound in the acceptor chamber relative to the initial donor concentration (3,12). It is well recognised that issues such as compound precipitation in donor buffer, non-specific adsorption to the diffusion apparatus, and high monolayer retention due to inadequate sink conditions (1,13,14) can invalidate key assumptions underpinning the calculation of P_app_ values by this approach (3,15). Alternative data analysis methods which account for some of these issues have been described (12,15,16); for example, high monolayer retention can be accounted for using a modified equation, provided the mass of compound retained in the cell monolayer has been measured. Alternatively, modified transport media have been employed, including the use of co-solvents, surfactants, complexation agents, bile salts, and proteins (17–20). These approaches require careful consideration of the potential impact of additives on membrane structure, tight junction integrity and transporter function as well as altered thermodynamic activity of the compound. Although these approaches have proven to be beneficial on a case-by-case basis, none can be considered to be generally applicable for compounds with differing physicochemical properties.

Several authors have also explored the applicability of serum binding proteins such as albumin or α_1_-acid glycoprotein (19,21,22). Inclusion of albumin in the basolateral chamber has been used as a means to better mimic *in vivo* conditions and maintain sink conditions (1,13), whilst having minimal impact on cell monolayer integrity, permeation and transporter function (23). However, when assessing permeability in the absorptive (apical-to-basolateral) direction, the presence of protein in the basolateral chamber alone has been reported to have minimal impact on compound recovery (21,24,25), and additional measures are required to minimise the impact of osmolality differences across the monolayer (26,27). Inclusion of protein in only one chamber also complicates the interpretation of bidirectional permeability data (24,28).

Herein, our aim was to assess the utility of human plasma as an assay medium in both donor and acceptor chambers of a Caco-2 cell monolayer test system, taking into account the unbound concentration in the donor chamber in the calculation of P_app_. While the use of human plasma in the basolateral chamber has been reported previously (29,30), the current studies were designed to explore the benefits of using human plasma simultaneously in both the apical and basolateral chambers such that conditions on each side of the monolayer are identical. Since protein binding in human plasma is usually measured during drug discovery, the calculation of P_app_ can readily incorporate the unbound donor concentration. In addition to improving sink conditions, the presence of plasma proteins in the apical chamber is likely to reduce non-specific adsorption and increase the apparent solubility leading to more accurate P_app_ measurements for poorly-soluble lipophilic molecules. In this study we utilised model compounds whose transmembrane flux occurs via either the paracellular or transcellular route, as well as compounds subject to P-gp-mediated efflux. In each category, compounds exhibiting low and high binding to human plasma proteins were included to probe (i) the impact of plasma on monolayer integrity, (ii) the effect of plasma on the apparent permeability of passively permeating molecules, (iii) the effect of plasma on the apparent efflux of P-gp substrates, and (iv) the ability of plasma to improve compound mass balance. The applicability of the approach was evaluated using a selected set of antimalarial drugs to support work being conducted using PBPK modelling. Many of these antimalarials showed very poor mass balance when permeability studies were conducted using traditional aqueous transport buffers thereby precluding the assessment of reliable permeability coefficients.

## Materials and Methods

### Materials

Human plasma was separated from whole blood obtained from the Australian Red Cross Blood Service and stored at −80°C. Caco-2 cells at passage 22 were purchased from American Type Culture Collection (ATCC; Rockville, MD). Buffer and cell culture components (including heat inactivated foetal bovine serum (FBS)), penicillin-streptomycin solution (containing 10,000 units/mL of penicillin and 10,000 μg/mL of streptomycin), and 0.25% trypsin-EDTA were from Life Technologies (Mulgrave, VIC, Australia). Corning cell culture consumables, HEPES buffer and selected test compounds (atenolol, cimetidine, ketoprofen, lucifer yellow, metoprolol, naproxen, paracetamol, propranolol, ranitidine, rhodamine 123 and verapamil) were from Sigma-Aldrich. Radiolabelled compounds (D-[1-^14^C]-mannitol and [^3^H(G)]-digoxin) were from Perkin-Elmer (Glen Waverley, VIC, Australia). Antimalarial compounds (amodiaquine, atovaquone, chloroquine, halofantrine, mefloquine, naphthoquine, piperaquine and quinine) were obtained from the Medicines for Malaria Venture (Geneva, Switzerland). Saquinavir was obtained from LGM Pharma (Nashville, TN) and talinolol was sourced from Cayman Chemicals (Ann Arbor, MI). All other reagents and solvents were of analytical grade.

### Caco-2 Cell Culture

Caco-2 cells (passage 30–40) were grown in flasks in an incubator (37°C, 5% CO_2_, 95% relative humidity) to approximately 80% confluence and treated with trypsin-EDTA solution before centrifugation and reconstitution in Dulbecco’s modified Eagle medium (DMEM; containing glutamax-1, D-glucose and sodium pyruvate) to a cell concentration of 500,000 cells/mL. Cells were then seeded onto polycarbonate membrane inserts (0.4 μm pore size, 0.33 cm^2^ area; Corning Incorporated, Corning, NY) at a density of 60,000 cells per well and allowed to differentiate and reach confluence over 22–24 days with media replenished every 2–3 days. Before each permeability assay, monolayer integrity was confirmed by measuring the TEER in transport buffer using an EVOM ohmmeter (World Precision Instruments, Sarasota, FL). Only monolayers with TEER values >200 Ω.cm^2^ were utilised.

### Permeability Assay

Experiments were performed using either aqueous transport buffer (Hanks balanced salt solution (HBSS) containing 20 mM HEPES, pH 7.4) or human plasma in both the apical and basolateral chambers. Donor solutions for non-radiolabelled compounds were prepared by spiking DMSO stock solutions into assay media (buffer or plasma) to achieve a typical compound concentration of between 10 μM and 30 μM. Concentrations outside of this range were used for propranolol (50 μM), compounds that exhibited low transmembrane flux (lucifer yellow, cimetidine, atenolol, ranitidine and rhodamine 123; 100 μM) or compounds with high plasma protein binding (ketoprofen and naproxen; 700 μM in plasma). For radiolabelled compounds, donor solutions were prepared at 1–2 μCi/mL for ^3^H-digoxin (total concentration of 0.034–0.067 μM) and 1.6 μCi/mL for ^14^C-mannitol (30 μM plus 50 μM unlabelled). For all compounds, a final DMSO concentration of 0.1% *v*/v was maintained in the donor medium. Donor solutions for non-radiolabelled compounds were equilibrated at 37°C for up to 4 h before centrifuging at 1300 *x g* for 5 min to remove any compound that may have precipitated. The visually clear supernate was then added to the donor chamber (*n* = 3 replicates for each direction), while the respective blank assay medium (i.e. buffer or plasma) was added to the acceptor chamber. The volume of assay medium in the apical and basolateral chambers was kept constant at 0.25 and 1.0 mL, respectively. During the transport assay, transwell plates were maintained at 37°C and shaken at 200 rpm using a THERMOstar plate incubator (BMG Labtech GmbH, Ortenberg, Germany).

Aliquots for the measurement of compound concentrations in the donor solution were taken at the start (approximately 1–2 min after addition to donor chamber) and end of the permeability experiment. Compound flux was determined by measuring concentrations in aliquots of the acceptor solution collected at the end of the transport experiment for lucifer yellow or periodically over 60–180 min for all other compounds. At each sample time, the volume of acceptor solution removed was replaced with an equal volume of blank assay medium (i.e. buffer or plasma) and acceptor concentrations were corrected for the dilution that occurred with media replacement. Independent experiments confirmed that the pH of plasma in the apical and basolateral chambers remained within the range of 7.4 ± 0.2 for approximately 120 min. The osmolality of both transport buffer and human plasma was ~310 mOsm/kg.

### Physicochemical Properties and Plasma Protein Binding

Physicochemical properties were calculated using Instant JChem (ver 16.4.11.0, ChemAxon, Budapest, Hungary). Binding was assessed using one of two methods: ultracentrifugation (31) using neat human plasma or rapid equilibrium dialysis (RED) using either neat or diluted plasma. For the ultracentrifugation method, human plasma was spiked with test compound (at a concentration comparable to that used in Caco-2 assay), vortex mixed briefly and aliquots (*n* = 3–4 per compound) transferred to ultracentrifuge tubes which were then allowed to equilibrate for 30 min at 37°C under a 5% CO_2_ atmosphere. These tubes were then transferred to the ultracentrifugation rotor (Beckman Rotor type 42.2 Ti) and maintained under the same CO_2_ atmosphere for a further 15 min prior to sealing the rotor and subjecting to ultracentrifugation (Beckman Optima XL-100 K; 223,000 *x g*) at 37°C for 4.2 h to separate proteins. Additional ultracentrifuge tubes containing spiked plasma were maintained at 37°C under 5% CO_2_ atmosphere for 0.5 h and 4.2 h to confirm stability and to measure the total plasma concentration. The pH of the plasma pre- and post-centrifugation was confirmed to be within 7.4 ± 0.1. To allow analysis with a single calibration curve, the total plasma and protein-free supernatant samples were ‘matrix-matched’ by taking aliquots of protein-free supernatant samples and adding to fresh tubes containing an equal volume of blank plasma, or taking aliquots of the total plasma samples and adding to fresh tubes containing an equal volume of Dulbecco’s phosphate-buffered saline (DPBS). In this way, the final composition of both the protein-free supernatant samples and the total plasma samples were ‘matched’ prior to the analysis against a common calibration curve prepared in a 1:1 mixture of human plasma and DPBS. All samples were then stored frozen at −80°C until analysis.

For the RED method, aliquots of neat plasma (*n* = 3 per compound spiked at a concentration comparable to that used in Caco-2 assay) and blank pH 7.4 phosphate buffered saline (PBS; 0.1 M sodium phosphate buffer containing 40 mM sodium chloride) were transferred to donor and dialysate chambers, respectively, of RED inserts (Thermo Scientific, Cat # 89810, MWCO ~8000) that were placed in a holding plate. The holding plate was then placed on a plate shaker (set at 800 rpm; ThermoMixer C, Eppendorf) and maintained at 37°C under a 2% CO_2_ atmosphere. The dialysis was conducted for 6 h, assuming steady state was achieved by the end of this period (32). Samples were then removed from both the donor and dialysate chambers of each RED insert. The post dialysis pH of plasma and dialysate buffer were confirmed to be within 7.4 ± 0.1. Samples were matrix matched as described above (but using PBS buffer instead of DPBS) and stored at −80°C until analysis.

For the more lipophilic compounds with potential for very high binding, the RED method was used with diluted plasma to increase the chance of detecting concentrations in the dialysate samples. Diluted human plasma (10% *v*/v in pH 7.4 phosphate buffered saline) was spiked with each compound (at 10% of the concentration used for the Caco-2 experiment with neat plasma) and binding conducted as described above. The binding in neat plasma was then calculated by correcting for the dilution as described below.

### Sample Analysis

For radiolabelled compounds (^14^C-mannitol and ^3^H-digoxin), donor and acceptor samples were mixed with 2 mL scintillation liquid (Ultima Gold, PerkinElmer, Downers Grove, IL) and the radioactivity in disintegrations per minute (DPM) measured using a Tri-Carb 2800 TR liquid scintillation analyser (PerkinElmer, Downers Grove, IL). The samples for lucifer yellow (λ_ex_ 430 nm, λ_em_ 535 nm) and rhodamine 123 (λ_ex_ 500 nm, λ_em_ 525 nm) were assayed by fluorescence using a FLUOstar plate reader (BMG Labtech GmbH, Ortenberg, Germany) against calibration standards prepared in the respective blank matrices. For all non-radiolabelled compounds, sample concentrations were measured using a Waters Micromass Quattro Premier, Waters Micromass Ultima PT, Waters Xevo TQ or a Waters Xevo TQD triple quadrupole mass spectrometer coupled to a Waters Acquity UPLC (Waters Corporation, Milford, MA). Chromatography was performed using a Supelco Ascentis Express RP Amide (50 × 2.1 mm, 2.7 μm) or Phenomenex Kinetex PFP (50 × 2.1 mm, 2.6 μm) column maintained at 40°C with mobile phase (A: 0.05% *v*/v formic acid or 0.005 M ammonium formate; B: acetonitrile or methanol) delivered by gradient elution. The mass spectrometer conditions were optimised for each compound under positive or negative mode electrospray ionisation and multiple-reaction-monitoring using specific *m/z* transitions, and with data acquisition performed using MassLynx software (see Supplementary Information; Tables S1-S3). Quantification of compound concentrations was achieved by comparison of peak response to that for calibration standards prepared in the equivalent blank matrix. All samples and calibration standards were processed in 96-well plates; proteins were precipitated using acetonitrile (3-fold volume ratio) containing diazepam (5 μg/mL; for assays using positive ionisation mode) or leucine enkephalin (10 μg/mL; for assays using negative ionisation mode) as the internal standard, and samples were centrifuged (21,000 *x g*) for 3 min at room temperature. Aliquots of the clear supernatants were transferred to fresh 96-well plates for analysis. Calibration curves were fit to a quadratic or linear equation as appropriate and assay runs were accepted if the accuracy (% deviation from the nominal concentration) was within ±15% (or ± 20% at the lower limit of quantitation) for the calibration standards and quality control (QC) samples, and precision (% relative standard deviation) was <15% for replicate QC samples.

### Data Analysis

For binding measurements in neat plasma by ultracentrifugation, the fraction unbound (*f*_*u*_) was calculated from the ratio of the average unbound concentration in protein free supernate (C_unbound_) to the average total concentration in non-centrifuged plasma (C_total_). The standard deviation (SD) for the *f*_*u*_ value was calculated using the propagation of errors approach as described previously (33). For binding measurements using the RED method in neat plasma, *f*_*u*_ was calculated from the ratio of the dialysate to total plasma concentration for each dialysis unit and the mean and SD calculated for replicate units (*n* = 3).

For RED binding assessments using 10% plasma, the *f*_*u* − *diluted*_ for individual dialysis units was calculated in the same way as described above. The *f*_*u* − *diluted*_ value was then corrected for the plasma dilution factor using eq. 1 (34) in which D is the dilution factor, and the mean and SD were then calculated for replicate dialysis units (*n* = 3):

1 $$ {f}_u=\frac{1/\mathrm{D}}{\left(\left(\frac{1}{fu- diluted}\right)-1\right)+1/\mathrm{D}} $$

In cases where compound concentrations in dialysate were below the analytical lower limit of quantification (LLOQ), a theoretical maximum *f*_*u*_ value was obtained using the LLOQ value and correcting for dilution using eq. 1.

For Caco-2 permeability experiments using buffer, the apparent permeability coefficient (P_app_) was calculated using eq. 2.

2 $$ {\mathrm{P}}_{\mathrm{app}}\ \left(\mathrm{cm}/\sec \right)=\frac{\mathrm{dQ}}{\mathrm{dt}}\times \frac{1}{{\mathrm{C}}_{\mathrm{donor}}^{\mathrm{initial}}\times \mathrm{A}} $$

where dQ/dt is the apparent steady-state rate of appearance (μmol/s) in the acceptor chamber based on the linear portion of the flux profile, A is the surface area of the monolayer (0.3 cm^2^) and ${\mathrm{C}}_{\mathrm{donor}}^{\mathrm{initial}}$ is the concentration in the donor chamber at the start of experiment (μmol/cm^3^).

Consistent with the approach reported previously for systems containing surfactant or binding protein in the donor chamber (29,35), P_app_ values for the plasma-based experiments were corrected for the unbound compound concentration in the donor chamber as per eq. 3.

3 $$ {\mathrm{P}}_{\mathrm{app}}\ \left(\mathrm{cm}/\sec \right)=\frac{\mathrm{dQ}}{\mathrm{dt}}\times \frac{1}{\left({\mathrm{C}}_{\mathrm{donor}}^{\mathrm{initial}}\times {\mathrm{f}}_{\mathrm{u}}\right)\times \mathrm{A}} $$

The mass balance at the end of the permeability experiment was obtained using eq. 4 where the mass of compound was calculated as the product of the molar concentration and volume of donor or acceptor solution:

4 $$ \mathrm{Mass}\ \mathrm{balance}\ \left(\%\right)=\frac{{\mathrm{Mass}}_{\mathrm{donor}}^{\mathrm{final}}+{\mathrm{Mass}}_{\mathrm{acceptor}}^{\mathrm{final}}}{{\mathrm{Mass}}_{\mathrm{donor}}^{\mathrm{initial}}}\times 100 $$

The apparent efflux ratio was obtained from the ratio of the mean P_app_ value in the basolateral to apical (B-A) relative to the apical to basolateral (A-B) direction. The standard deviation (SD) for the apparent efflux ratio was calculated using the propagation of errors approach as described above.

The relationship between the apparent permeability coefficient (P_app_) and the fraction absorbed in humans was evaluated by fitting a sigmoidal function to the data:

5 $$ FA=\frac{100\ast {P}_{app}^{\gamma }}{P_{app}^{\gamma }+{P}_{app50}^{\gamma }} $$

where FA is human fraction absorbed based on reports in the literature (see Table I), P_app_ is the apparent Caco-2 permeability coefficient, and P_app50_ is the permeability coefficient associated with 50% fraction absorbed. Permeability classifications of low (less than 50% absorption in humans), intermediate (50–84% absorption) or high (>85% absorption) were based on the FDA Guidance for Industry (7).

**Table I:** Calculated Physicochemical Parameters, Measured Human Plasma Protein Binding and Human Fraction Absorbed Data for Compounds Included in the Study

Compound type	Compound	MW	H-bond D/A ^a^	PSA (Å^2^) ^b^	cLog P	cLogD_7.4_	pKa ^c^	f_u_^d^	Human FA (%)^f^
Passive paracellular	Lucifer yellow	444.9	6/11	239	−5.03	−6.76	–	0.51	0
	^14^C-Mannitol	182.2	6/6	121	−3.73	−3.73	–	0.83	26
	Cimetidine	252.3	3/5	89	−0.11	−0.22	–	0.76	64
	Atenolol	266.3	3/4	85	0.43	−1.80	9.7	0.77	55
	Ranitidine	314.4	2/5	84	0.99	0.45	7.8	0.92	50
Passive transcellular	Metoprolol	267.4	2/4	50.7	1.76	−0.47	9.7	0.70	95
	Naproxen	230.3	1/3	46.5	2.99	−0.05	4.2*	0.018	100
	Propranolol	259.3	2/3	41.5	2.58	0.36	9.7	0.25	100
	Ketoprofen	254.3	1/3	54.4	3.61	0.39	3.9*	0.023	100
	Paracetamol	151.2	2/2	49.3	0.91	0.90	–	0.86	90
P-gp substrates	Talinolol	363.5	4/4	82.6	2.80	0.49	9.8	0.48	40
	^3^H-Digoxin	780.9	6/13	203	2.37	1.92	7.2*	0.61	81
	Saquinavir	670.8	5/7	167	3.16	2.05	8.5	0.040	30
	Verapamil	454.6	0/6	63.9	5.04	2.79	9.7	0.14	100
	Rhodamine 123	344.4	2/4	85.4	2.85	2.37	7.7	0.35	na
Antimalarial compounds	Chloroquine	319.9	1/3	28.2	3.93	0.88	10.3	0.61	na
	Quinine	324.4	1/4	45.6	2.51	0.86	9.0	0.37 ^e^	na
	Amodiaquine	355.9	2/4	48.4	3.80	2.32	10.2	0.12	na
	Naphthoquine	410.0	3/4	57.2	5.22	3.35	10.6	0.019 ^e^	na
	Mefloquine	378.3	2/3	45.2	4.11	2.07	9.5	0.015 ^e^	na
	Piperaquine	535.5	0/6	38.7	5.27	3.87	7.4, 8.5	<0.001 ^e^	na
	Atovaquone	366.8	1/3	54.4	5.00	3.33	5.7*	<0.0002 ^e^	na
	Halofantrine	500.4	1/2	23.5	8.06	5.46	10.1	<0.0001 ^e^	na

^a^Number of hydrogen bond donors (D)/acceptors (A)

^b^Total polar surface area at pH 7.4

^c^Only basic pKa values >7.4 and acidic pKa values (*) of <7.4 were included

^d^Fraction unbound in plasma

^e^f_u_ determined using diluted plasma with correction for dilution as described in the methods

^f^Human fraction absorbed (FA) values from the literature: paracetamol, cimetidine, atenolol, ranitidine (42); ketoprofen, naproxen, metoprolol, propranolol, verapamil (43); mannitol (44); saquinavir (45); and lucifer yellow (46). na = not available

### Statistics

Mass balance, permeability coefficients and efflux ratios using buffer and plasma were compared using an unpaired t-test testing for significance at α = 0.05.

## Results

### Plasma Protein Binding and Physicochemical Properties

Measured plasma protein binding values along with calculated physicochemical properties are shown in Table I. A measurable f_u_ value was obtained for all compounds except piperaquine, atovaquone and halofantrine for which no measurable concentrations were detected in dialysate samples, even using diluted plasma. Compounds ranged in protein binding from very low (f_u_ > 0.9) to very high (f_u_ < 0.0001). Compound binding was assessed at a similar concentration to that used in the donor chamber for permeability assessment to take into account the potential for saturable binding at high concentration.

### Bidirectional Permeability and Efflux Ratios for Passively Permeating Compounds

**Fig. 1:**
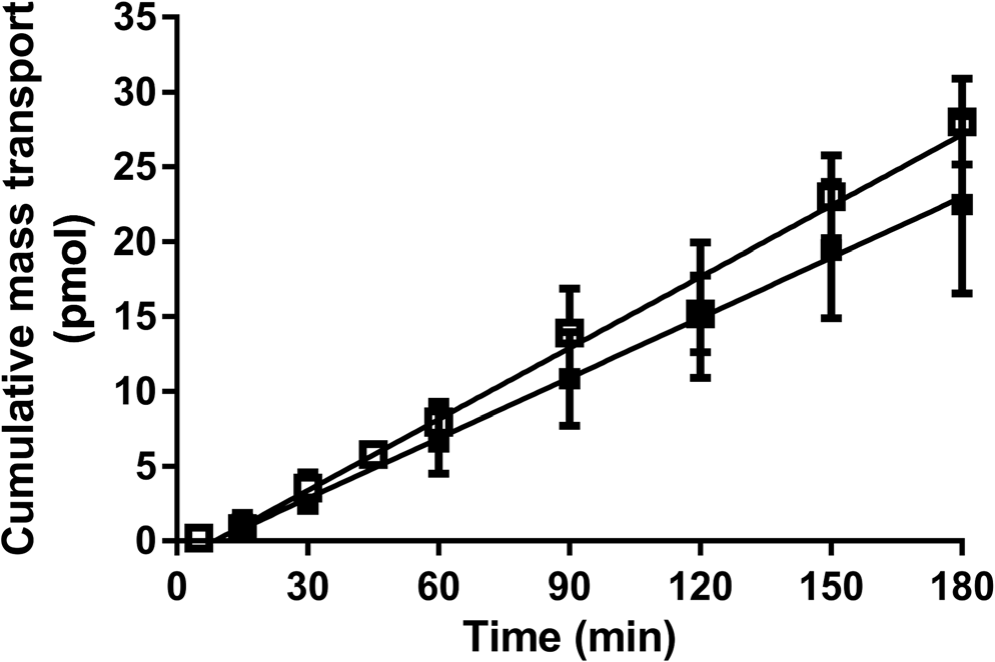
A-B Flux profiles for ^14^C-Mannitol in buffer (open squares) *vs* plasma (closed squares) over a 180 min sampling period (mean ± SD, *n* = 3).

Bidirectional permeability was assessed for selected passively permeating compounds in both buffer and plasma (Table II). Each of the two compounds in this set exhibited good mass balance using each medium and differences between the two media were not statistically significant. As expected, mannitol (low protein binding), showed only minor differences in the P_app_ or efflux ratio between the two media (Table II and Fig. 1). For propranolol (high protein binding), the P_app_ in both directions was higher in plasma, however the efflux ratio was unaffected by the medium.

**Table II:** Bidirectional Permeability Data in Buffer and Plasma for Passively Permeating Compounds. Data Represent the Mean ± SD for n = 3 Flux Profiles Per Experiment

Compound (f_u_ in plasma)^a^	Assay media	Mass balance (%)		P_app_ (10^−6^ cm/s)		Efflux ratio
		A-B	B-A	A-B	B-A	
^14^C-Mannitol (0.83)	Buffer	99 ± 2.3	99 ± 1.7	0.29 ± 0.051	0.56 ± 0.026	1.9 ± 0.35
	Plasma	96 ± 4.0	97 ± 5.2	0.31 ± 0.081	0.43 ± 0.021^b^	1.4 ± 0.37
Propranolol (0.25)	Buffer	85 ± 7.8	97 ± 0.58	58 ± 4.5	52 ± 4.0	0.9 ± 0.10
	Plasma	97 ± 4.6	99 ± 2.3	120 ± 35^b^	110 ± 19^b^	0.9 ± 0.31

^a^f_u_ values determined at a similar concentration compared to that used for the Caco-2 experiment

^b^value in plasma was statistically different to the corresponding value in buffer (α = 0.05)

### Apical to Basolateral Permeability for Passively Permeating Compounds

Mass balance and A-B permeability data for additional passively permeating compounds are shown in Table III. For the paracellular compounds, two (atenolol and ranitidine) showed an increase in P_app_ with plasma that was statistically significant, whereas the other two (lucifer yellow and cimetidine) showed no difference. For the passive transcellular compounds, the low binding compounds (paracetamol and metoprolol) showed very similar P_app_ values using the two media (differences not significant) while the higher binding compounds (propranolol, ketoprofen and naproxen) exhibited 2- to 3-fold higher P_app_ using plasma as the medium compared to buffer, although the increase for ketoprofen was not statistically significant.

**Table III:** A-B Permeability Data in Buffer and Plasma for Passively Permeating Compounds. Data Represent the Mean ± SD for *n* = 3 Flux Profiles Per Experiment

Compound (f_u_ in plasma)^a^	Assay media	Mass balance (%)	P_app_ (10^−6^ cm/s)
		A-B	A-B
Lucifer yellow (0.51)	Buffer	86 ± 6.7	0.21 ± 0.085
	Plasma	98 ± 4.7	0.17 ± 0.069
Atenolol (0.77)	Buffer	82 ± 4.9	0.39 ± 0.051
	Plasma	84 ± 4.0	1.1 ± 0.10^b^
Cimetidine (0.76)	Buffer	94 ± 2.9	1.7 ± 0.45
	Plasma	97 ± 9.5	1.9 ± 0.26
Ranitidine (0.92)	Buffer	97 ± 11	0.99 ± 0.11
	Plasma	92 ± 7.2	2.1 ± 0.058^b^
Paracetamol (0.86)	Buffer	92 ± 3.8	32 ± 2.9
	Plasma	92 ± 0.58	37 ± 4.0
Metoprolol (0.70)	Buffer	83 ± 8.0	50 ± 6.1
	Plasma	85 ± 2.0	57 ± 5.8
Ketoprofen (0.023)	Buffer	95 ± 5.1	31 ± 6.7
	Plasma	88 ± 1.5	62 ± 19
Naproxen (0.018)	Buffer	89 ± 7.1	40 ± 7.0
	Plasma	85 ± 3.1	130 ± 36^b^

^a^f_u_ values determined at a similar concentration compared to that used for the Caco-2 experiment

^b^value in plasma was statistically different to the corresponding value in buffer (α = 0.05)

### Bidirectional Permeability and Efflux Ratios for P-Gp Substrates

**Fig. 2:**
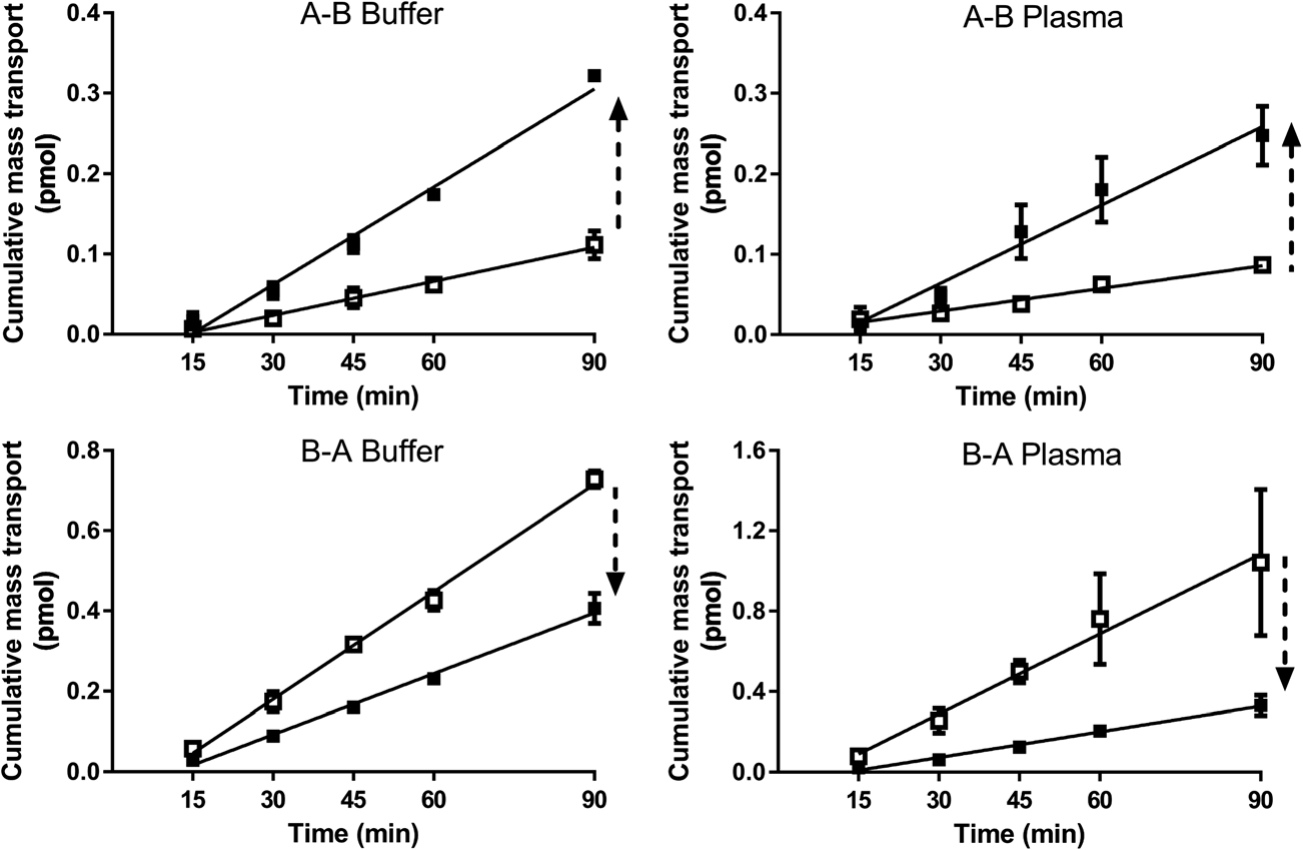
Bidirectional flux profiles for ^3^H-digoxin in buffer and plasma in the absence (open squares) and presence (closed squares) of 100 μM verapamil (mean ± SD, *n* = 3).

Among the P-gp substrates (Table IV), mass balance was high for all compounds and did not differ significantly between the two media. The only exceptions were verapamil (for B-A only) and saquinavir (for both A-B and B-A) for which there was a significant increase in mass balance in going from buffer to plasma. For these moderate to highly bound compounds, P_app_ values generally increased in both directions when plasma was used as the medium compared to buffer, although in a few cases (B-A for digoxin, A-B for digoxin + verapamil) the increases were not statistically significant. The one exception was rhodamine 123 which showed no difference in the A-B P_app_ between the two media. The increase in P_app_ values for digoxin in plasma *versus* buffer was evident both in the absence and presence of verapamil (as a P-gp inhibitor). For most compounds, efflux ratios remained comparable for the two media in spite of the increase in permeability in the presence of plasma. In the case of digoxin there was a marginal (but statistically significant) decrease in the efflux ratio, whereas for rhodamine 123, the efflux ratio increased in the presence of plasma. In the presence of verapamil, the efflux ratio for digoxin reduced to approximately one in both buffer and plasma (Fig. 2).

**Table IV:** Bidirectional Permeability Data in Buffer and Plasma for P-gp Substrates. Data Represent the Mean ± SD for n = 3–4 Flux Profiles Per Experiment

Compound (f_u_ in plasma)^a^	Assay media	Mass balance (%)		P_app_ (10^−6^ cm/s)		Efflux ratio
		A-B	B-A	A-B	B-A	
^3^H-Digoxin (0.61)	Buffer	100 ± 0	100 ± 0	2.7 ± 0.21	18 ± 0.58	6.7 ± 0.56
	Plasma	93 ± 7.6	100 ± 5.8	4.7 ± 0.15^b^	26 ± 1.5	5.5 ± 0.36^b^
^3^H-Digoxin (0.61) + 100 μM Verapamil	Buffer	99 ± 0.58	100 ± 0.58	4.6 ± 0.46	5.8 ± 0.53	1.3 ± 0.18
	Plasma	96 ± 2.1	99 ± 0.58	6.2 ± 1.0	7.4 ± 0.49^b^	1.2 ± 0.21
Talinolol (0.48)	Buffer	99 ± 1.7	100 ± 6.1	1.1 ± 0.058	17 ± 1.5	15 ± 1.5
	Plasma	96 ± 1.2	98 ± 2.1	2.8 ± 0.46^b^	34 ± 2.6^b^	12 ± 2.2
Rhodamine 123 (0.35)	Buffer	99 ± 11	99 ± 1.2	1.6 ± 0.32	9.7 ± 1.1	6.1 ± 1.4
	Plasma	98 ± 1.5	92 ± 4.7	1.2 ± 0.058	14 ± 1.5^b^	12 ± 1.4^b^
Verapamil (0.14)	Buffer	91 ± 8.5	83 ± 5.8	41 ± 4.6	45 ± 1.5	1.1 ± 0.13
	Plasma	94 ± 2.1	96 ± 5.5^b^	110 ± 12^b^	120 ± 5.8^b^	1.1 ± 0.13
Saquinavir (0.040)	Buffer	76 ± 0.58	60 ± 5.6	2.4 ± 1.4	34 ± 2.1	14 ± 8.2
	Plasma	100 ± 7.2^b^	110 ± 7.5^b^	8.4 ± 2.5^b^	140 ± 42^b^	17 ± 7.2

^a^f_u_ values determined at a similar concentration compared to that used for the Caco-2 experiment

^b^value in plasma was statistically different to the corresponding value in buffer (α = 0.05)

### Relationship between Fraction Absorbed in Humans and Caco-2 Permeability Using Buffer and Plasma

**Fig. 3:**
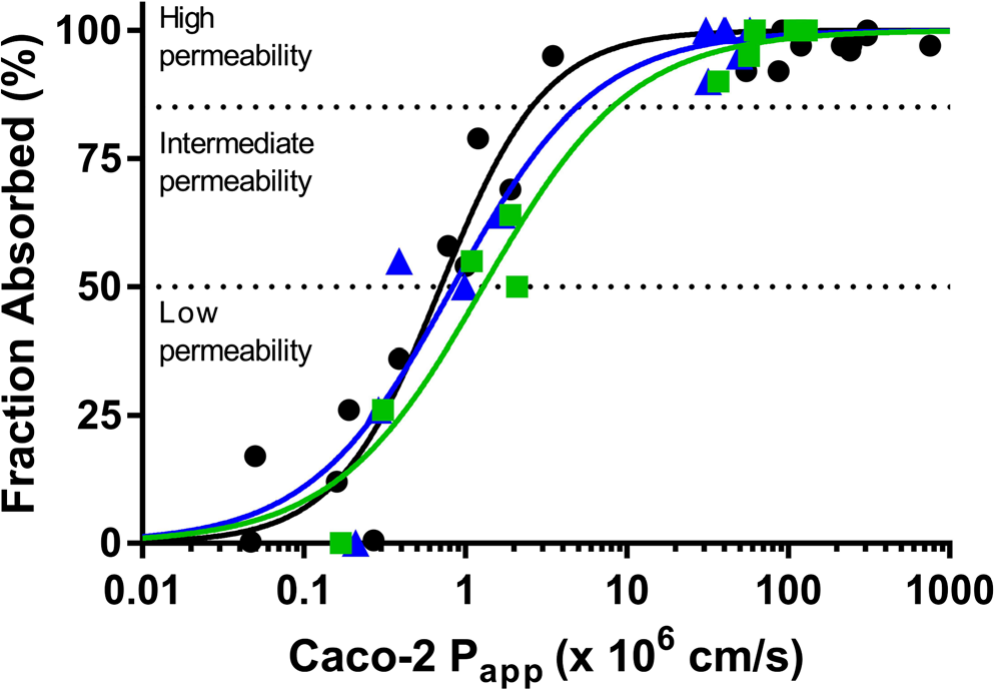
Relationship between Caco-2 cell permeability values using pH 7.4 buffer (blue) or human plasma (green) assay media and human fraction absorbed values from the literature (Table I). Blue symbols are P_app_ data using pH 7.4 buffer, green symbols are data obtained using human plasma and black symbols are data from Stenberg *et al*. using pH 7.4 buffer (36). High, intermediate and low permeability classifications are based on (7). Regression parameters are shown in Table VI. Caco-2 P_app_ values represent the mean of 3 measurements however error bars have been omitted for clarity.

Recognising that the widespread use of the Caco-2 cell monolayer system is based largely on a general positive correlation between Caco-2 P_app_ and human jejunal permeability and fraction absorbed (2,3), we assessed the relationship between the Caco-2 P_app_ values and human fraction absorbed based on the available data (Fig. 3 and Table V). Although our dataset is smaller, the trends for the buffer P_app_ values are reasonably consistent with results reported previously (36). For P_app_ values determined in plasma, the profile is shifted to higher P_app_ values consistent with the general trend for higher permeability for many of the compounds tested with plasma.

**Table V:** Relationship between Caco-2 Permeability Values for Passively Permeating Compounds Obtained using Either pH 7.4 Buffer or Human Plasma and Human Fraction Absorbed Values. P_app50_ is the Permeability Coefficient Associated with 50% Fraction Absorbed and γ is the Hill Slope. Regression Parameters Represent the Best Fit to a Sigmoidal Equation ± Standard Error of the Estimate

	Regression Parameters (P_app_ X 10^6^ cm/s)	Permeability Classification^a^ (P_app_ X 10^6^ cm/s)	
Caco-2 P_app_ (pH 7.4 buffer) This work	P_app50_ 0.839 ± 0.202 γ 0.982 ± 0.259	Low Int High	P_app_ < 0.839 0.839 ≤ P_app_ < 4.90 P_app_ ≥ 4.90
Caco-2 P_app_ (human plasma) This work	P_app50_ 1.29 ± 0.213 γ 0.942 ± 0.173	Low Int High	P_app_ < 1.29 1.29 ≤ P_app_ < 8.15 P_app_ ≥ 8.15
Caco-2 P_app_ (pH 7.4 Buffer) Stenberg *et al*. (36)	P_app50_ 0.705 ± 0.079 γ 1.34 ± 0.200	Low Int High	P_app_ < 0.705 0.705 ≤ P_app_ < 2.57 P_app_ ≥ 2.57

^a^Using the criteria of low (FA <50%), intermediate (FA 50–84%), and high (FA ≥ 85%) as per reference (7)

### Bidirectional Permeability of Compounds Exhibiting Poor Mass Balance

**Fig. 4:**
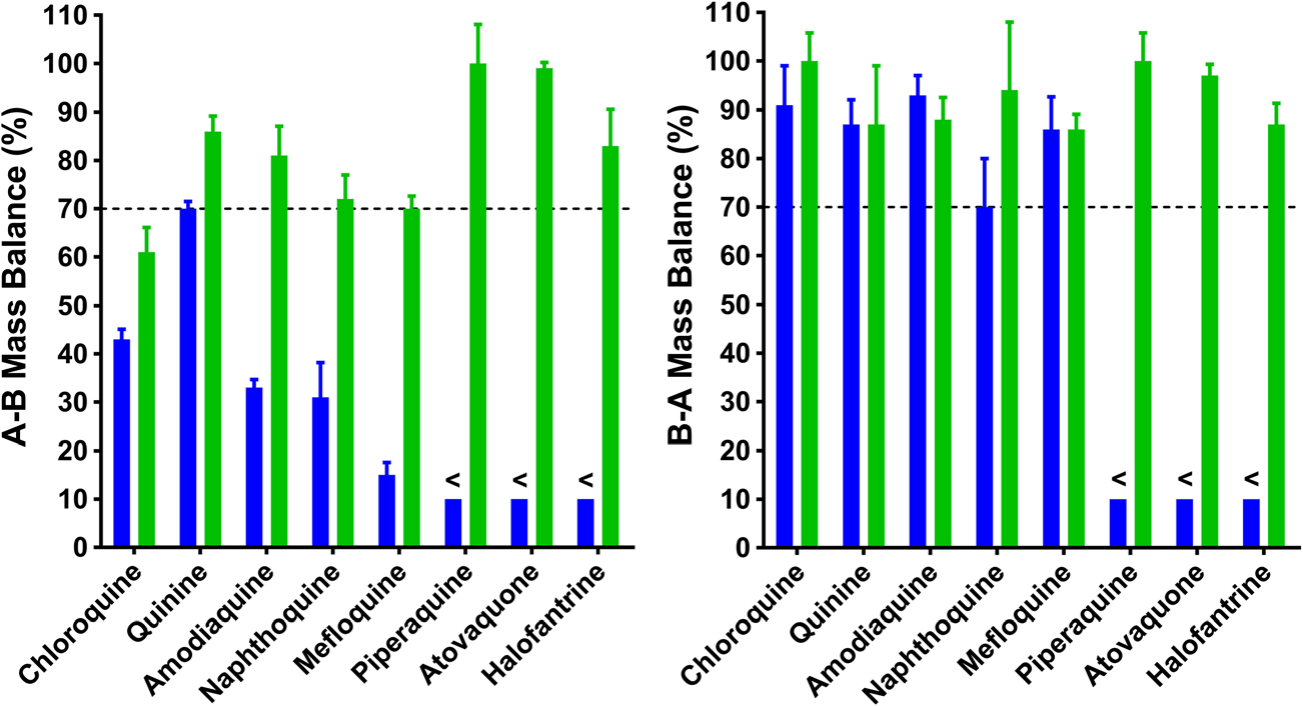
Mass balance data for selected antimalarial compounds using either buffer (blue bars) or plasma (green bars) as the assay medium. Bars represent the mean ± SD for *n* = 3 replicates. The dashed line represents a nominal target minimum mass balance level of 70%. Less than signs (<) represent a mass balance value of <10% in buffer.

Additional studies were conducted using a set of antimalarial compounds that demonstrated moderate to poor mass balance when buffer was used as the assay medium. As shown in Table VI and Fig. 4, most compounds showed a marked increase in mass balance using plasma as the medium. Three compounds, including chloroquine, napthoquine, mefloquine, showed an increase in mass balance with plasma but only to approximately 60–70%.

**Table VI:** Bidirectional Permeability Data in Buffer and Plasma for Selected Antimalarial Compounds. Data Represent the Mean ± SD for n = 3 Flux Profiles Per Experiment

Compound (f_u_ in plasma^a^)	Assay media	Mass balance (%)		P_app_ (10^−6^ cm/s)		Efflux ratio	Permeability Classification
		A-B	B-A	A-B	B-A		
Chloroquine (0.61)	Buffer	43 ± 2.1	91 ± 8.0	9.7 ± 0.46	19 ± 2.1	2.0 ± 0.24	High
	Plasma	61 ± 5.1^b^	110 ± 5.8^b^	16 ± 2.5^b^	22 ± 4.6	1.4 ± 0.37	High
Quinine (0.37)	Buffer	70 ± 1.5	87 ± 5.1	39 ± 5.0	40 ± 4.5	1.0 ± 0.17	High
	Plasma	86 ± 3.2^b^	87 ± 12	35 ± 4.2	57 ± 4.2^b^	1.6 ± 0.23^b^	High
Amodiaquine (0.12)	Buffer	33 ± 1.7	93 ± 4.0	4.5 ± 0.12	7.1 ± 1.9	1.6 ± 0.43	Intermediate
	Plasma	81 ± 6.1^b^	88 ± 4.6	90 ± 8.3^b^	63 ± 9.6^b^	0.7 ± 0.12^b^	High
Naphthoquine (0.019)	Buffer	31 ± 7.2	70 ± 10	2.2 ± 1.3	4.5 ± 1.4	2.0 ± 1.3	Intermediate
	Plasma	72 ± 5.0^b^	94 ± 14	230 ± 32^b^	180 ± 10^b^	0.78 ± 0.12	High
Mefloquine (0.015)	Buffer	15 ± 2.6	86 ± 6.7	2.1 ± 0.65	4.7 ± 0.35	2.2 ± 0.70	Intermediate
	Plasma	70 ± 2.6^b^	86 ± 3.1	180 ± 20^b^	150 ± 10^b^	0.83 ± 0.11^b^	High
Piperaquine (<0.001)	Buffer	<10	<10	c.n.d.	c.n.d.	c.n.d.	Low
	Plasma	100 ± 8.1	100 ± 5.8	>300	>300	c.n.d.	High
Atovaquone (<0.0002)	Buffer	<10	<10	c.n.d	c.n.d.	c.n.d.	Low
	Plasma	99 ± 1.2	97 ± 2.3	>300	>300	c.n.d.	High
Halofantrine (<0.0001)	Buffer	<10	<10	c.n.d.	c.n.d.	c.n.d.	Low
	Plasma	83 ± 7.6	87 ± 4.4	>300	>300	c.n.d.	High

c.n.d. Could not determine as no measurable concentrations were detected in acceptor chamber

^a^f_u_ values determined at a similar concentration compared to that used for the Caco-2 experiment

^b^value in plasma was statistically different to the corresponding value in buffer (α = 0.05)

Calculated P_app_ values for the antimalarials in both A-B and B-A directions generally increased when plasma was used as the assay medium with only a few exceptions (chloroquine B-A P_app_ and quinine A-B P_app_). Permeability classifications changed from being either low or intermediate with buffer, to high using plasma for all compounds except chloroquine and quinine which were classified as being high in both media.

## Discussion

The usefulness of *in vitro* permeability measurements hinges on the ability to generate reliable P_app_ values. Permeability assessment of lipophilic compounds is often plagued by low mass balance due to inadequate solubility in the aqueous transport media, non-specific adsorption to the transwell apparatus or high monolayer retention (through a variety of different mechanisms), all leading to inaccurate P_app_ estimates (1). To address these issues, we explored the utility of human plasma as an alternative assay medium in the Caco-2 permeability assay. The potential advantages of this system are improved compound solubilization, reduced non-specific adsorption, and reduced monolayer retention due to improved sink conditions. Furthermore, human plasma protein binding is generally measured during drug discovery allowing the unbound concentration in the donor chamber to be easily accounted for in the calculation of P_app_. In these studies, binding was assessed at a concentration comparable to that used for the permeability experiment to avoid the potential for saturable binding for those compounds that were assessed at high concentration.

When using plasma as the assay medium, the change in compound flux across the cell monolayer (relative to that using buffer as the medium) will be dictated by the interplay between the reduced free compound concentration in the donor medium (which reduces trans-monolayer flux) and increased compound desorption into the acceptor medium due to improved sink conditions (which increases trans-monolayer flux). In the present study, the P_app_ values generated using plasma were corrected for the fraction unbound to account for the impact of the unbound donor concentration on compound flux. As such, differences in P_app_ between buffer and plasma result from changes in adsorption to the apparatus and/or compound desorption from the monolayer into the acceptor medium, provided any impact of plasma on monolayer integrity and transporter function can be ruled out.

To determine the impact of plasma on monolayer integrity, five low to intermediate permeability compounds (mannitol, lucifer yellow, atenolol, cimetidine, and ranitidine) were assessed using buffer and plasma as the medium in both the apical and basolateral chambers (Tables II and III). As expected, each of these compounds showed high mass balance (>80%) in both directions (where assessed) and in both media. For mannitol, the flux profile remained linear over 180 min with both media (Fig. 1) suggesting the absence of changes to monolayer integrity over an extended time period. In general, there were no major changes in the A-B P_app_ values for this set of compounds, except for atenolol and ranitidine where P_app_ increased but still remained very low.

The impact of plasma on transcellular permeation was then evaluated using low (paracetamol and metoprolol) and high (propranolol, ketoprofen and naproxen) binding compounds (Tables II and III). Each of these compounds had high mass balance in both media. For both paracetamol and metoprolol, the P_app_ values in the two assay media were similar confirming that plasma had minimal impact on compound desorption into the acceptor solution for these low binding compounds. In contrast, propranolol, ketoprofen and naproxen each exhibited a 2- to 3-fold increase in P_app_ using plasma as the medium. As there were no mass balance issues for these compounds, the increased P_app_ with plasma is likely due to improved desorption of compound from the monolayer into the acceptor medium because of enhanced sink conditions. These results are similar to previous observations with various lipophilic compounds when purified proteins were included in the acceptor medium (17,21).

Another aim of these investigations was to determine the effect of plasma on P-gp mediated efflux. This was evaluated using known P-gp substrates of varying lipophilicity and protein binding (Table I). Verapamil, a known high permeability P-gp substrate which shows no net-efflux (37,38), exhibited efflux ratios close to unity in both media, however it showed higher P_app_ in plasma (Table IV), consistent with the data for propranolol (Table II). For compounds known to exhibit polarised flux, four compounds with moderate to high binding (digoxin, talinolol, rhodamine 123 and saquinavir) were evaluated. For digoxin and talinolol (both moderately bound), P_app_ was higher in plasma than in buffer although the increase in B-A P_app_ for digoxin was not statistically significant and as a result the efflux ratio was reduced (Table IV). The inclusion of verapamil as a competitive P-gp inhibitor resulted in the expected increase in digoxin A-B P_app_ and decrease in B-A P_app_ with the resulting efflux ratio being close to unity. The effect of P-gp inhibition on A-B and B-A permeability of digoxin in the presence of verapamil was also evident in the measured flux profiles prior to applying the binding correction (Fig. 2). In the case of saquinavir, mass balance increased significantly with plasma, and the bidirectional P_app_ values using plasma were also higher than the values using buffer, however the efflux ratio was unchanged between the two media (Table IV).

Distinct from the other efflux markers, rhodamine 123 exhibited a (statistically significant) higher efflux ratio with plasma (Table IV). This was primarily due to an increased B-A P_app_ in plasma, as the A-B P_app_ was largely similar for both media. Previous reports have suggested that the absorptive flux of rhodamine 123 occurs largely via the paracellular route (39), therefore the lack of a significant sink effect in the A-B direction may not be surprising. Overall, these data suggest that plasma had no adverse impact on P-gp mediated efflux and that the efflux ratios remain largely similar when bidirectional transport occurs via the transcellular route.

Having established that the use of plasma had no negative impact on monolayer integrity, transcellular permeation or P-gp mediated efflux, the key objective of this work was to improve mass balance and sink conditions for a set of relatively lipophilic antimalarial compounds for which reliable P_app_ values could not be determined using buffer as the medium. This work was part of a larger collaborative project with the Medicines for Malaria Venture to assess *in vitro* ADME properties of a set of legacy antimalarial drugs for use in PBPK modelling. For the eight antimalarial compounds examined, seven were weak bases and one was a weak acid. Calculated Log D_pH 7.4_ values ranged from 0.86 to 5.46 and f_u_ values ranged from 0.541 to <0.0001 (Table I). When assessed using buffer, the mass balance in the A-B direction was low (<45%) for all compounds except quinine which had a mass balance of ~70% (Table VI). For three compounds (halofantrine, atovaquone and piperaquine), mass balance using buffer was <10% in both the A-B and B-A directions (Table VI). For the remaining six compounds, mass balance using buffer in the A-B direction was low (15–40%) but each exhibited good mass balance in the B-A direction (>70%). Direction-dependent differences in cellular retention have been described by others where it was proposed to be related to direction dependent differences in barrier properties although data to support this hypothesis were not presented (24,40).

When assessed using plasma as the assay medium, the mass balance for all compounds increased to ≥60% in both the A-B and B-A directions (Table VI and Fig. 4). The impact of plasma was most dramatic for halofantrine, atovaquone and piperaquine, where the determination of P_app_ values in buffer was precluded by the absence of measurable concentrations in the acceptor solution. In contrast, all three compounds exhibited well-defined flux profiles when assessed using plasma. While chloroquine, naphthoquine, and mefloquine each showed improved mass balance in the presence of plasma, mass balance was still only about 60–70% in the A-B direction. We cannot rule out a contribution of lysosomal sequestration as has previously been described for chloroquine (41).

All of the antimalarial compounds tested exhibit physicochemical properties that would suggest that they should have high membrane permeability (Table I). Using buffer as the assay medium, and ignoring the impact of low mass balance and/or ineffective sink conditions, all compounds except for quinine and chloroquine would be classified as having low or intermediate permeability on the basis of the measured P_app_ values (Table VI). Using plasma as the assay medium, the bidirectional P_app_ values for the five compounds where f_u_ could be determined, ranged from approximately 16 to 230 × 10^−6^ cm/s (Table VI). For piperaquine, atovaquone and halofantrine, where very high binding precluded the measurement of f_u_, the bidirectional P_app_ values were based on theoretical minimum f_u_ values and were estimated to be very high (> 300 × 10^−6^ cm/s). Therefore, using plasma as the assay medium, measured or approximated P_app_ values for each of these compounds was high, a result which is far more in line with their calculated physicochemical properties.

The use of plasma proteins in the basolateral chamber has been reported previously by others (29,30) and has been shown to increase permeability in the absorptive direction. However the inclusion of protein in the basolateral chamber only is unlikely to overcome mass balance issues resulting from poor solubility and/or adsorption in the apical chamber (21). Furthermore, using protein-based media in the basolateral chamber only complicates the interpretation of bidirectional permeability data (28) and can lead to an imbalance in the osmolality on each side of the membrane (26,27). Using plasma on both sides of the membrane addresses each of these issues, thereby providing improved sink conditions and improving compound recovery.

For this method to be effectively utilized, plasma protein binding needs to be measured in parallel studies which can be challenging for the type of compounds likely to benefit from this approach. For such compounds, special care has to be taken in choosing an appropriate assay method to minimize the impact of non-specific adsorption and/or lipoprotein association which can confound protein binding measurements. Another potential challenge is that the reduced thermodynamic activity resulting from binding to proteins in the donor plasma means that the cumulative mass of compound appearing in the acceptor chamber is reduced, placing heavy demands on analytical sensitivity. This can generally be overcome by running the Caco-2 assay at higher donor concentrations provided binding is also assessed at the same concentration to rule out the potential for saturable protein binding.

## Conclusions

The results presented here illustrate the potential advantages of using plasma as the assay medium for lipophilic compounds which are prone to non-specific adsorption and/or high monolayer retention. Correcting permeability coefficients for the fraction unbound provides a means of accounting for the reduced thermodynamic activity that results from binding to plasma proteins. The results demonstrated that there is no negative impact of plasma on membrane integrity, transcellular permeation or P-gp-mediated efflux and that for highly lipophilic compounds, mass balance is significantly improved leading to more reliable estimation of the permeability coefficient. The method provided a way of assessing the permeability of a set of highly lipophilic antimalarial compounds leading to permeability classifications that are consistent with their physicochemical properties.

## Electronic supplementary material

ESM 1 (DOCX 38 kb)

## Abbreviations

DMEM: Dulbecco’s Modified Eagle Medium
DMSO: Dimethylsulfoxide
DPBS: Dulbecco’s Phosphate-Buffered Saline
HEPES: 2-[4-(2-hydroxyethyl)piperazin-1-yl]ethanesulfonic acid
P_app_: Apparent permeability coefficient
P-gp: P-glycoprotein
TEER: Transepithelial electrical resistance
