# Supplementary material for: Using Human Plasma as an Assay Medium in Caco-2 Studies Improves Mass Balance for Lipophilic Compounds
Source: Pharm Res. 2018 Sep 17;35(11):210. doi: 10.1007/s11095-018-2493-3 (PMC6156755; doi:10.1007/s11095-018-2493-3)
Supplement: Supplementary file 1 — (DOCX 38 kb) [file 11095_2018_2493_MOESM1_ESM.docx]

**SUPPLEMENTARY INFORMATION**

**Table S1** LC conditions used for analysis of plasma protein binding and Caco-2 permeability samples.

| **Compound** | **LC Column*** | **Mobile Phase**  **(Organic / Aqueous)** | **Flow rate**  **(mL/min)** | **Gradient (% organic)** | | | | | | **Cycle time** |
| --- | --- | --- | --- | --- | --- | --- | --- | --- | --- | --- |
|  |  |  |  | **Initial** | **Step 1** | **Step 2** | **Step 3** | **Step 4** | **Step 5** |  |
| Cimetidine | 1 | Acetonitrile/water with 0.05% formic acid | 0.4 | 0%  0-0.2 min | to 10%  0.2-1 min | to 95%  1-2.7 min | to 0%  3.3-3.5 min | 0%  3.5-4 min | --- | 4 min |
| Atenolol | 1 | Acetonitrile/water with 0.05% formic acid | 0.4 | 0%  0-0.2 min | to 10%  0.2-1 min | to 95%  1-2.7 | to 0%  3.3-3.5 min | 0%  3.5-4 min | --- | 4 min |
| Ranitidine | 2 | Acetonitrile/water with 0.05% formic acid | 0.4 | 0%  0-1 min | to 30%  1-1.5 min | to 95%  1.5-2.7 min | to 0%  3.3-3.5 min | 0%  3.5-4 min | --- | 4 min |
| Metoprolol  (buffer) | 1 | Acetonitrile/water with 0.05% formic acid | 0.4 | 2%  0-0.2 min | to 10%  0.2-0.3 min | to 95%  0.3-2.7 min | to 2%  3.3-3.5 min | 2%  3.5-4 min | --- | 4 min |
| Metoprolol  (plasma) | 1 | Acetonitrile/water with 0.05% formic acid | 0.4 | 2%  0-0.2 min | to 5%  0.2-0.3 min | to 80%  0.3-2.7 min | to 95%  2.7-2.8 min | to 2%  3.3-3.5 min | 2%  3.5-4 min | 4 min |
| Naproxen | 1 | Acetonitrile/0.005 M ammonium formate with 0.05% formic acid | 0.4 | 2%  0-0.2 min | to 10%  0.2-0.3 min | to 95%  0.3-2.7 min | to 2%  3.3-3.5 min | 2%  3.5-4 min | --- | 4 min |
| Propranolol | 1 | Acetonitrile/water with 0.05% formic acid | 0.4 | 2%  0-0.2 min | to 5%  0.2-0.3 min | to 60%  0.3-2.7 min | to 95%  2.7-2.8 min | to 2%  3.3-3.5 min | 2%  3.5-4 min | 4 min |
| Ketoprofen | 1 | Acetonitrile/0.005 M ammonium formate with 0.05% formic acid | 0.4 | 2%  0-0.2 min | to 10%  0.2-0.3 min | to 95%  0.3-2.7 min | to 2%  3.3-3.5 min | 2%  3.5-4 min | --- | 4 min |
| Paracetamol | 1 | Acetonitrile/water with 0.05% formic acid | 0.4 | 0%  0-0.2 min | to 10%  0.2-0.3 min | to 80%  0.3-2.7 min | to 95%  2.7-2.8 min | to 0%  3.3-3.5 min | 0%  3.5-4 min | 4 min |
| Talinolol | 1 | Acetonitrile/water with 0.05% formic acid | 0.4 | 1%  0-0.3 min | to 70%  0.3-2.7 min | to 95%  2.7-2.8 min | to 1%  3.3-3.5 min | 1%  3.5-4 min | --- | 4 min |
| Saquinavir  (buffer) | 1 | Acetonitrile/water with 0.05% formic acid | 0.4 | 0.1%  0-1 min | to 95%  1-2.7 min | to 0.1%  3.3-3.5 min | 0.1%  3.5-4 min | --- | --- | 4 min |
| Saquinavir  (plasma) | 1 | Acetonitrile/water with 0.05% formic acid | 0.4 | 0.2%  0-0.2min | to 0.5%  0.2-0.3min | to 70%  0.3-2.7 min | to 95%  2.7-2.8 min | to 0.2%  3.3-3.5 min | 0.2%  3.5-4 min | 4 min |
| Verapamil | 1 | Acetonitrile/water with 0.05% formic acid | 0.4 | 2%  0-0.2 min | to 10%  0.2-0.3 min | to 90%  0.3-2.7 min | to 95%  2.7-2.8 min | to 2%  3.3-3.5 min | 2%  3.5-4 min | 4 min |
| Chloroquine | 1 | Acetonitrile/water with 0.05% formic acid | 0.4 | 0%  0-0.3 min | to 70%  0.3-2.7 min | to 95%  2.7-2.8 min | to 0%  3.3-3.5 min | 0%  3.5-4 min | --- | 4 min |
| Quinine | 1 | Acetonitrile/water with 0.05% formic acid | 0.4 | 2%  0-0.2 min | to 5%  0.2-0.3 min | to 60%  0.3-2.7min | to 95%  2.7-2.8 min | to 2%  3.3-3.5 min | 2%  3.5-4 min | 4 min |
| Amodiaquine  (buffer) | 1 | Acetonitrile/water with 0.05% formic acid | 0.4 | 0%  0-0.3 min | to 80%  0.3-2.7 min | to 95%  2.7-2.8 min | to 0%  3.3-3.5 min | 0%  3.5-4 min | --- | 4 min |
| Amodiaquine  (plasma) | 1 | Acetonitrile/water with 0.05% formic acid | 0.4 | 0.1%  0-0.3 min | to 60%  0.3-2.7 min | to 95%  2.7-2.8 min | to 0.1%  3.3-3.5 min | 0.1%  3.5-4 min | --- | 4 min |
| Naphthoquine  (buffer) | 1 | Acetonitrile/water with 0.05% formic acid | 0.4 | 2%  0-0.2 min | to 10%  0.2-0.3 min | to 60%  0.3-2.7 min | to 95%  2.7-2.8 min | to 2%  3.3-3.5 min | 2%  3.5-4 min | 4 min |
| Naphthoquine  (plasma) | 1 | Acetonitrile/water with 0.05% formic acid | 0.4 | 0.1%  0-0.3 min | to 60%  0.3-2.7 min | to 95%  2.7-2.8 min | to 0.1%  3.3-3.5 min | 0.1%  3.5-4 min | --- | 4 min |
| Mefloquine | 1 | Acetonitrile/water with 0.05% formic acid | 0.4 | 2%  0-0.2 min | to 10%  0.2-0.3 min | to 60%  0.3-2.7 min | to 95%  2.7-2.8 min | to 2%  3.3-3.5 min | 2%  3.5-4 min | 4 min |
| Piperaquine  (buffer) | 1 | Acetonitrile/0.005 M ammonium formate with 0.05% formic acid | 0.4 | 0%  0-0.3 min | to 5%  0.3-1.0 min | to 95%  1.0-2.7 min | to 0%  3.3-3.5 min | 0%  3.5-4 min | --- | 4 min |
| Piperaquine  (plasma) | 1 | Acetonitrile/0.005 M ammonium formate with 0.05% formic acid | 0.4 | 2%  0-0.2 min | to 50%  0.2-0.3 min | to 95%  0.3-2.7 min | to 2%  3.3-3.5 min | 2%  3.5-4 min | --- | 4 min |
| Atovaquone  (buffer) | 1 | Methanol/0.005 M ammonium formate | 0.4 | 2%  0-0.2 min | to 30%  0.2-0.3 min | to 95%  0.3-2.7 min | to 2%  3.3-3.5 min | 2%  3.5-4 min | --- | 4 min |
| Atovaquone  (plasma) | 1 | Methanol/0.005 M ammonium formate | 0.4 | 2%  0-0.2 min | to 60%  0.2-0.3 min | to 95%  0.3-2.7 min | to 2%  3.3-3.5 min | 2%  3.5-4 min | --- | 4 min |
| Halofantrine | 1 | Acetonitrile/water with 0.05% formic acid | 0.4 | 2%  0-0.2 min | to 20%  0.2-0.3 min | to 95%  0.3-2.7 min | to 2%  3.3-3.5 min | 2%  3.5-4 min | --- | 4 min |

*LC column 1: Supelco Ascentis Express RP Amide (50 x 2.1 mm, 2.7 µm); LC column 2: Phenomenex Kinetex PFP (50 x 2.1 mm, 2.6 µm)

**Table S2** Mass spectrometer conditions used for analysis of plasma protein binding and Caco-2 permeability samples.

| **Compound** | **Detection mode** | **MRM (m/z)** | **Cone voltage (V)** | **CID (V)** |
| --- | --- | --- | --- | --- |
| Cimetidine | Positive electrospray ionisation | 253.25 > 159.17 | 30 | 15 |
| Atenolol | Positive electrospray ionisation | 267.27 > 145.07 | 40 | 25 |
| Ranitidine | Positive electrospray ionisation | 315.31 > 176.07 | 30 | 15 |
| Metoprolol | Positive electrospray ionisation | 268.40 > 116.03 | 35 | 20 |
| Naproxen | Negative electrospray ionisation | 229.06 > 185.08 | 15 | 7 |
| Propranolol | Positive electrospray ionisation | 259.96 > 116.09 | 30-40 | 30 |
| Ketoprofen | Negative electrospray ionisation | 253.07 > 209.10 | 20 | 8 |
| Paracetamol | Positive electrospray ionisation | 151.85 > 109.85 | 30-35 | 15 |
| Talinolol | Positive electrospray ionisation | 364.26 > 308.18 | 40 | 30 |
| Saquinavir | Positive electrospray ionisation | 671.31 > 570.22 | 60 | 50 |
| Verapamil | Positive electrospray ionisation | 455.30 > 165.15 | 50 | 30 |
| Chloroquine | Positive electrospray ionisation | 320.24 > 247.14 | 35 | 20 |
| Quinine | Positive electrospray ionisation | 325.28 > 81.08 | 40 | 25 |
| Amodiaquine | Positive electrospray ionisation | 356.18 > 283.00 | 35 | 15-25 |
| Naphthoquine | Positive electrospray ionisation | 410.16 > 337.2 | 30-50 | 20-35 |
| Mefloquine | Positive electrospray ionisation | 379.20 > 361.18 | 40 | 25 |
| Piperaquine (buffer) | Positive electrospray ionisation | 535.24 > 69.99 | 45 | 35 |
| Piperaquine (plasma) | Positive electrospray ionisation | 535.15 > 288.11 | 45 | 35 |
| Atovaquone | Positive electrospray ionisation | 365.09 > 336.96 | 45-50 | 30-45 |
| Halofantrine | Positive electrospray ionisation | 500.30 > 142.08 | 35 | 25 |

**Table S3** Assay validation data for LC-MS analysis of plasma protein binding and Caco-2 permeability samples.

| **Compound** | **Assay Matrix** | **Accuracy**  **(%)** | **Precision**  **(% RSD)** | **Calibration range (ng/mL)** | **LLQ**  **(ng/mL)** |
| --- | --- | --- | --- | --- | --- |
| Cimetidine | Buffer | ± 4.4 | <7.7 | 0.5 - 10,000 | 5.0 |
|  | Plasma | ± 2.5 | <6.9 | 0.5 - 10,000 | 5.0 |
| Atenolol | Buffer | ± 9.7 | <7.7 | 0.5 - 10,000 | 5.0 |
|  | Plasma | ± 2.8 | <8.1 | 0.5 - 10,000 | 5.0 |
| Ranitidine | Buffer | ± 4.8 | <7.8 | 0.5 - 20,000 | 5.0 |
|  | Plasma | ± 0.2 | <2.6 | 0.5 - 20,000 | 5.0 |
| Metoprolol | Buffer | ± 6.2 | <2.5 | 0.5 – 10,000 | 0.5 |
|  | Plasma | ± 8.6 | <9.3 | 0.5 – 10,000 | 1.0 |
| Naproxen | Buffer | ± 3.6 | <14.3 | 0.5 – 10,000 | 10 |
|  | Plasma | ± 1.6 | < 8.0 | 50 – 20,000 | 50 |
| Propranolol | Buffer | ± 8.4 | < 3.8 | 0.5 - 5,000 | 10.0 |
|  | Plasma | ± 4.6 | < 2.5 | 0.5 - 5,000 | 0.5 |
| Ketoprofen | Buffer | ± 7.2 | < 6.2 | 0.5 – 10,000 | 50 |
|  | Plasma | ± 5.1 | < 8.9 | 50 – 100,000 | 100 |
| Paracetamol | Buffer | ± 8.4 | < 8.9 | 0.5 – 10,000 | 1.0 |
|  | Plasma | ± 8.6 | < 14.2 | 0.5 – 10,000 | 5.0 |
| Talinolol | Buffer | ± 4.3 | < 5.3 | 0.5 – 10,000 | 5.0 |
|  | Plasma | ± 2.4 | < 7.7 | 0.5 – 10,000 | 0.5 |
| Saquinavir | Buffer | ± 10.8 | < 6.4 | 0.5 – 2,000 | 0.5 |
|  | Plasma | ± 10.5 | < 7.6 | 0.5 – 5,000 | 0.5 |
| Verapamil | Buffer | ± 6.3 | < 3.4 | 0.5 – 10,000 | 0.5 |
|  | Plasma | ± 8.4 | < 3.3 | 0.5 – 10,000 | 5.0 |
| Chloroquine | Buffer | ± 2.7 | < 6.7 | 0.5 – 10,000 | 5.0 |
|  | Plasma | ± 7.9 | < 9.9 | 0.5 – 2,000 | 5.0 |
| Quinine | Buffer | ± 1.7 | < 7.8 | 0.5 – 10,000 | 5.0 |
|  | Plasma | ± 7.4 | < 5.7 | 0.5 – 10,000 | 5.0 |
| Amodiaquine | Buffer | ± 7% | < 12% | 0.5 – 10,000 | 5.0 |
|  | Plasma | ± 12% | < 7% | 0.5 – 10,000 | 0.5 |
| Naphthoquine | Buffer | ± 8.1% | < 6.4% | 0.5 – 10,000 | 0.5 |
|  | Plasma | ± 4.5% | < 6.7% | 0.5 - 5000 | 0.5 |
| Mefloquine | Buffer | ± 7.0% | < 9.8% | 0.5 – 10,000 | 0.5 |
|  | Plasma | ± 2.2% | < 5.0% | 0.5 – 10,000 | 0.5 |
| Piperaquine | Buffer | ± 2.9% | < 11.7% | 0.5 – 10,000 | 1.0 |
|  | Plasma | ± 6.7% | < 7.1% | 0.5 – 10,000 | 50 |
| Atovaquone | Buffer | ± 12.9% | < 6.0% | 0.5 – 10,000 | 5.0 |
|  | Plasma | ± 5.3% | < 5.4% | 0.5 – 5,000 | 5.0 |
| Halofantrine | Buffer | ± 0.9% | < 4.5% | 0.5 – 10,000 | 1.0 |
|  | Plasma | ± 10.2% | < 8.9% | 0.5 – 2,000 | 0.5 |

LLQ – Lower Limit of Quantitation
